# Balloon-assisted Transcatheter arterial embolization using N-butyl cyanoacrylate for iatrogenic arterial bleeding by groin puncture: a new technology

**DOI:** 10.1186/s42155-020-00132-3

**Published:** 2020-08-23

**Authors:** Tatsuo Ueda, Satoru Murata, Hidemasa Saito, Izumi Miki, Daisuke Yasui, Fumie Sugihara, Wataru Shimizu, Shin-Ichiro Kumita

**Affiliations:** 1 Department of Radiology, Nippon Medical School, 1-1-5 Sendagi, Bunkyo-ku, Tokyo, 113-8603 Japan; 2 Center for Interventional Radiology, Teikyo University Chiba Medical Center, 3426-3 Anesaki, Ichihara-City, Chiba, 299-0011 Japan; 3 Department of Cardiovascular Medicine, Nippon Medical School, 1-1-5 Sendagi, Bunkyo-ku, Tokyo, 113-8603 Japan

**Keywords:** Balloon-assisted, Iatrogenic arterial bleeding, Groin puncture, N-butyl cyanoacrylate, Trans-arterial embolization

## Abstract

**Background:**

Balloon-assisted transcatheter arterial embolization (TAE) using n-butyl cyanoacrylate (NBCA) and lipiodol (Lp) mixture is a new endovascular treatment technique for iatrogenic arterial bleeding by groin puncture. It is less invasive compared to surgical repair, and NBCA migration into the circulation can be prevented by temporary balloon occlusion of the parent artery without ultrasound-guidance. This study aimed to report on the technical aspects and evaluate the efficacy and safety of fluoroscopically guided balloon-assisted transcatheter arterial embolization using NBCA for iatrogenic arterial bleeding by groin puncture.

**Materials and methods:**

The study included five patients (mean age 54.6 years; 3 male and 2 female) with iatrogenic arterial bleeding by groin puncture. We performed transcatheter arterial embolization using NBCA while occluding the responsible artery with a balloon catheter during the embolization to prevent NBCA migration. Two sheaths were inserted into the common femoral artery. A microcatheter was advanced into the pseudoaneurysm or extravasation via the contralateral sheath. A balloon catheter was advanced into the responsible artery until the balloon portion covered the leakage site via another sheath. After balloon inflation, the NBCA and Lip mixture was slowly injected until the pseudoaneurysm, or the extravasation was filled without touching the balloon. The microcatheter was removed immediately after the filling. We assessed technical success, overall success, and complications.

**Results:**

The injured arteries were the external iliac artery (*n* = 1), the common femoral artery (*n* = 2), and the proximal portion of the superficial femoral artery (n = 2). NBCA was injected once in four cases and twice in one case where complete hemostasis could not be achieved with one injection. The technical and overall success rate was 100% with no complications, including distal embolization of NBCA.

**Conclusions:**

Balloon-assisted TAE using NBCA is a feasible, effective, and safe treatment for iatrogenic arterial bleeding by groin puncture. It may also be applicable in other arterial bleeding situations where the potential risk of distal embolization can be decreased by applying the balloon-assisted technique.

## Background

The reported incidence of femoral post-catheterization pseudoaneurysms after coronary angioplasty and general intervention is 2–8% and 1.1%, respectively (Katzenschlager et al. 1995; Lumsden et al. 1999). Although ultrasound-guided compression is a simple and less invasive treatment for iatrogenic femoral arterial bleeding, this method has its disadvantages, including prolonged procedure time, discomfort for patients, and recurrence risk (0–20%) (Liau et al. 1997). Traditionally, surgical repair is the standard management method in failed cases of ultrasound-guided compression. Ultrasound-guided thrombin injection (UGTI), developed in recent years, has a relatively high success rate (92–98%) and has been the first-line choice nowadays (Vlachou et al. 2011). However, 17–23% of patients who underwent the procedure required more than one injection (Paulson et al. 2000). In addition, it can cause acute lower limb ischemia due to the escape of thrombin into the circulation despite with or without balloon protection of the neck. (Bhat and Chakraverty 2007; Paulson et al. 2001).

Balloon-assisted transcatheter arterial embolization (TAE) using an n-butyl cyanoacrylate (NBCA) and lipiodol (Lp) mixture is a new endovascular treatment technique for iatrogenic arterial bleeding by groin puncture. It is less invasive compared to surgical repair, and NBCA migration into the circulation can be prevented by temporary balloon occlusion of the parent artery without ultrasound-guidance. Therefore, this new technique may be an alternative option for pseudoaneurysms where thrombin injection is failed or risky.

This study aimed to report the technical aspects of the procedure and evaluate the efficacy and safety of fluoroscopically guided balloon-assisted TAE using NBCA for iatrogenic arterial bleeding by groin puncture.

## Materials and methods

### Patients

The study involved five patients (3 male and 2 female) who underwent balloon-assisted TAE using NBCA for iatrogenic arterial bleeding by groin puncture between June 2015 and April 2017. Four of the patients who presented with pseudoaneurysms were first treated with ultrasound-guided compression. However, this treatment failed in all cases. The fifth patient who presented with extravasation of contrast medium could not undergo ultrasound-guided compression because of active bleeding into the retroperitoneum. This study was approved by the institutional review board of our university hospital; informed consent was obtained from all patients before treatment commencement.

### Endovascular procedure

Two sheaths (4–6 Fr Supersheath, Medikit, Tokyo, Japan) were inserted into the contralateral common femoral artery for this procedure in four cases. In the other case, two 8Fr sheaths used in a previous catheter procedure were left in-situ in each side of common femoral arteries at the beginning of the treatment. A 4F guide catheter (C-MRT, Medikit, Tokyo, Japan) was inserted via the contralateral sheath. A 1.9F microcatheter (Goldcrest neo; Goldcrest medic, Tokyo, Japan) was advanced into the aneurysm or the extravasation via the guide catheter with the aid of a 0.018-in. micro-guidewire (Aqua VIII; Cardinal Health Japan, Tokyo, Japan). A 6, 8, or 10 mm diameter by 40 mm length balloon catheter (Mustang, Boston Scientific Japan, Tokyo, Japan) was advanced into the responsible artery until the balloon portion covered the site of leakage via another sheath with the aid of a 0.035-in. guidewire (Radifocus Guide Wire M; Terumo, Tokyo, Japan). The balloon diameter was selected depending on the diameter of the common femoral artery. NBCA (NBCA, Histoacryl, B. Braun AG, Melsungen, Germany) was prepared as a liquid embolic material by mixing NBCA with Lp (iodinated ethyl esters of the fatty acid of poppy seed containing 40% iodine; Lp, Gerbe, France) using a three-way stopcock. The balloon catheter was inflated across the injured portion of the vessel prior to the NBCA-Lp injection. After the balloon inflation, the lumen of the microcatheter was flushed with a 5% dextrose solution (OTSUKA GLUCOSE INJECTION; Otsuka, Naruto, Japan) to avoid adhesion to the catheter. Subsequently, NBCA-Lp was slowly injected until the pseudoaneurysm or extravasation was filled without touching the balloon. The movement of NBCA-Lip was monitored fluoroscopically in real-time to avoid touching the balloon. The microcatheter was removed immediately after the filling. The balloon inflation was maintained for 30 s. Angiography was conducted following deflation of the balloon to confirm the absence of blood flow in the pseudoaneurysm or extravasation, and to exclude distal NBCA migration. The patients were instructed to lie flat for 6 h after the procedure.

### Assessment of endovascular treatment efficacy

We assessed technical success, overall success, and complications. Technical success was defined as the disappearance of pseudoaneurysm or extravasation by final arteriography. Overall success was defined as complete hemostasis during the follow-up. Complications were defined as significant complications that required additional treatment. Follow-up studies were performed using ultrasound and enhanced CT.

## Results

### Patient characteristics

Patient characteristics are presented in Table 1.

**Table 1:** Backgrounds of patients

Case	Age	Gender	Symptoms	Catheter procedure	Anticoagulant, Antiplatelet, and/or Thrombolytic therapy	Imaging diagnosis	Imaging finding	Responsible artery	Size of PA (mm)	Neck diameter of PA (mm)	Interval from onset (days)
**1**	52	F	Groin distension, Pain	Neuro intervention	Heparin, Urokinase	Enhanced CT	Retroperitoneal hemorrhage with extravasation	Rt EIA	–	–	1
**2**	55	M	Groin distension, Pain	RFCA	Rivaroxaban	Enhanced CT	PA	Lt SFA	14	2	within 1 (8 h)
**3**	69	F	Groin distension, Pain	RFCA	Warfarin	Enhanced CT	PA	Lt SFA	21	3	2
**4**	50	M	Groin distension, Pain	Neuro intervention	Heparin, Aspirin, Clopidogrel	Enhanced CT	PA	Rt CFA	26	3	2
**5**	47	M	Groin distension, Pain	Coronary intervention	Clopidogrel	Arteriography	PA	Rt CFA	16	2	Within 1 (6 h)

*RFCA* Radiofrequency catheter ablation, *CT* Computed tomography, *PA* Pseudoaneurysm,

*Rt* Right, *Lt* Left, *EIA* External iliac artery, *CFA* Common femoral artery, *SFA* Superficial femoral artery

### Procedure results

**Fig. 1:**
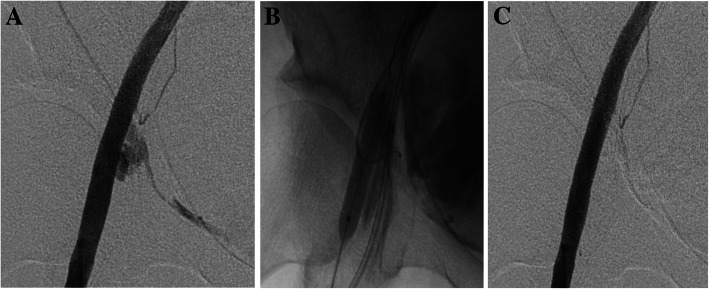
52-year-old female patient with right external iliac arterial (EIA) bleeding. **a** Pre-treatment arteriogram demonstrates active extravasation from the right EIA just below the inguinal ligament level. **b** A microcatheter was advanced into the extravasation and injection of 1.0 ml of the NBCA-Lp mixture (ratio of NBCA to Lp; 1:2) was performed via the microcatheter under an 8-mm-diameter × 4-cm-length angioplasty balloon inflation. **c** No extravasation of contrast medium is seen on the arteriography performed immediately after treatment

The procedure results are presented in Table 2. Case 1, which had a sheath left in the ipsilateral common femoral artery, showed extravasation in the distal site of the external iliac artery (Fig. 1a). NBCA was mixed with Lp at a ratio of 1:2 (*n* = 4) or 1:3 (*n* = 1). In case 5, we injected NBCA-Lp twice at the same procedure because the arteriography revealed that complete hemostasis could not be achieved after the first injection. In the other four cases, NBCA-Lip was injected only once (Fig. 1b). The total volume range of injected NBCA-Lp was 0.5–1.0 ml. In all the patients, arteriography showed the disappearance of the pseudoaneurysm or extravasation just after the embolization (Fig. 1c). The mean follow-up duration after the procedure was 7.9 ± 5.0 months (range: 0.5–13 months). Post-operative CT follow-up was performed for all patients, and the CT revealed no recurrence of bleeding in all cases. The technical and overall success rate in all the patients was 100%. There were no complications, including migration of NBCA-Lip following deflation of the balloon catheter and resultant limb ischemia.

**Table 2:** Procedure results

Case	Approach	Sheath size (Fr)	Balloon size (mm)	Ratio of NBCA to Lp	Injection volume of NBCA-Lp (ml)	Number of injections	Complications	Follow-up duration (months)	Follow-up CT (days after treatment)
1	Bil CFA	8, 8	8	1:2	1	1	None	0.5	15
2	Rt CFA × 2	4, 6	6	1:2	0.5	1	None	7	7
3	Rt CFA × 2	4, 5	6	1:2	0.5	1	None	13	2
4	Lt CFA × 2	4, 6	10	1:3	1	1	None	12	4
5	Lt CFA × 2	4, 6	10	1:2	0.5, 0.3	2	None	7	5

*Bil* Bilateral, *Rt* Right, *Lt* Left, *CFA* Common femoral artery, *NBCA* N-Butyl Cyanoacrylate, *Lp* Lipiodol

## Discussion

We treated iatrogenic arterial bleeding by groin puncture using the new technique of balloon-assisted TAE with NBCA and obtained a 100% technical and overall success rate without any complications. Although NBCA is often used for trans-arterial embolization of acute arterial bleeding (Kish et al. 2004), it is difficult to use in parent arterial bleeding because it has a potential risk of peripheral organ ischemia due to distal embolization of NBCA. Balloon-assisted TAE could prevent the migration of NBCA, thereby mitigating against distal embolization. Another advantage of the present technique is that the movement of NBCA-Lip can be monitored fluoroscopically in real-time. We could stop the NBCA injection properly by monitoring fluoroscopically in real-time, thus reducing the risk of NBCA adhering to the balloon catheter and promoting the reliable treatment of the pseudoaneurysm or extravasation. Moreover, ultrasound guidance is not necessary for this procedure. Ultrasound-guided procedures are difficult to perform in cases where the bleeding point is an intrapelvic or a visceral artery. Therefore, we believe that this technique may apply to arterial bleeding other than that of the femoral artery. We did not experience any adhesion of NBCA to the balloon catheter and microcatheter because we injected NBCA carefully to avoid it touching the balloon catheter. However, we believe there is a small potential risk of the NBCA adhering to the balloon catheter in this procedure. Tanaka et al. reported that the balloon catheter and microcatheter adhered strongly to the vessel in balloon-assisted packing of wide-neck aneurysms with NBCA-Lp mixture in an experimental study (Tanaka et al. 2015). In our study, the neck of the pseudoaneurysms was narrow (2–3 mm). This may partly explain why we did not experience any adhesion of the NBCA. Nevertheless, we believe the most important requirement in this procedure is to inject NBCA carefully without touching the balloon catheter. Nakai et al. reported percutaneous fluoroscopically guided NBCA injection for iatrogenic femoral arterial pseudoaneurysm under temporary balloon occlusion of arterial blood flow (Nakai et al. 2012). Although this technique is similar to our technique, it requires ultrasound guidance to puncture the pseudoaneurysm, which is different from our technique. Therefore, it may not be applicable in arteries that cannot be visualized by ultrasound.

The limitation of the technique is that it requires two punctures of the femoral artery due to the use of a microcatheter and a balloon catheter. This increases the possibility of new iatrogenic arterial bleeding. Additionally, it may be technically difficult to cannulate a microcatheter into the bleeding point of an aneurysm with a very narrow neck. Furthermore, this technique was only used in pseudoaneurysms with a narrow neck, and it might be needed as an adjunctive technique that allows the treatment of wide-necked pseudoaneurysms.

## Conclusion

In conclusion, balloon-assisted TAE using NBCA could be a feasible, effective, and safe treatment modality for iatrogenic arterial bleeding by groin puncture as an alternative treatment of ultrasound-guided thrombin injection. It may also be applicable in another arterial bleeding such as visceral or limb arteries, where the potential risk of distal embolization can be prevented by applying the balloon-assisted technique.

## Data Availability

Data sharing is not applicable to this article as no datasets were generated or analyzed during the current study.

## Abbreviations

TAE: Assisted transcatheter arterial embolization
NBCA: N-butyl cyanoacrylate (NBCA)
Lp: Lipiodol
